# FOSTERING DEMENTIA-FRIENDLY COMMUNITIES AND SUPPORTING CAREGIVERS THROUGH THE GWEP PROGRAMS

**DOI:** 10.1093/geroni/igz038.1363

**Published:** 2019-11-08

**Authors:** Jennifer J Severance, Lisa O’Neil, Mindy Fain

**Affiliations:** 1 University of North Texas Health Science Center, Fort Worth, Texas, United States; 2 University of Arizona, Tuscon, Arizona, United States; 3 University of Arizona, Tucson, Arizona, United States

## Abstract

As the number of people affected by dementia increases, it is essential that caregivers are provided resources and communities educated on how to engage and support people living with dementia. Supporting caregivers and providing education about dementia-friendly communities are key components of the Geriatric Workforce Enhancement Programs. This symposium will provide examples of GWEP projects which foster dementia-friendly communities and support caregivers. Additionally, recent and current legislation targeting dementia and caregivers will be presented.

